# Editorial: Molecular regulation of tumor cells migration and metastatic growth

**DOI:** 10.3389/fonc.2023.1329053

**Published:** 2023-11-17

**Authors:** Michelle L. Matter, Vasiliki Gkretsi

**Affiliations:** ^1^ School of Medicine, University of Tulane, New Orleans, LA, United States; ^2^ Louisiana Cancer Research Center, New Orleans, LA, United States; ^3^ Biomedical Sciences Program, Department of Life Sciences, School of Sciences, European University Cyprus, Nicosia, Cyprus; ^4^ Cancer Metastasis and Adhesion Group, Basic and Translational Cancer Research Center (BTCRC), European University Cyprus, Nicosia, Cyprus

**Keywords:** migration, invasion, metastasis, miRNAs, EMT, uPA, cytoskeleton

## Migration and metastasis in cancer

The migratory and invasive capacity of cells is the key differentiating factor between benign and malignant tumors that affects patient prognosis and survival, as more than 90% of cancer deaths is due to metastasis. Hence, metastasis is a complex multistep process involving gene and signaling pathway changes that lead to cancer cell migration, invasion, and ultimately establishment of a new metastatic tumor. The collection of articles in this *Frontiers* R*esearch* T*opic* presents recent advances and reviews current knowledge in the molecular regulation of malignant transformation driving tumor cell migration and metastatic growth.

Cell adhesion and migration are essential for malignant transformation, cancer progression and the development of chemoresistance. Cancer cells hijack various molecular pathways to evade cell regulatory constraints. For example, cancer cells utilize multiple migration and invasion methods during metastasis. The switch between migration/invasion modes is called invasion plasticity and is the topic of the review by Legatova et al. Rapid modulation in cell morphology due to changes in microtubules and cross talk with cytoskeletal networks are critical components in metastasis and tumor cell plasticity. Remodeling of the actin cytoskeleton is another way tumor cells transition to the malignant phenotype. Wang et al. review how actin cytoskeleton remodeling is regulated by actin-binding proteins with a focus on the LIM domain and acting-binding protein (LIMA_1_). They discuss how LIMA_1_ dysregulation contributes to changes in cytoskeletal dynamics promoting cancer cell migration and invasion. Indeed, changes at the leading edge of tumor cells alters migration and cytoskeletal dynamics. Original research by Asano et al. identifies that lamellipodia formation in migrating cells is controlled by conversion of inositol phospholipid PI(4,5)P_2_ into PI(3,4,5)P_3_. In particular, these authors demonstrate cancer cell migration is regulated by VIPR2, a vasoactive receptor for vasoactive intestinal peptide (VIP), which controls actin nucleation and lamellipodium formation via PI(3,4,5)P_3_. Another cytoskeletal interacting protein cytoplasmic Sirtuin 1 (SIRT1) interacts with cortactin to promote cancer cell migration and metastasis. This member of the Sirtuins family is a histone deacetylase enzyme, which requires the nicotinamide adenine dinucleotide (NAD^+^) co-factor to function. Nuclear SITR1 deacetylates several oncogenes and transcription factors to control their function. Ahmad et al. review SITR1 function and its role as a novel target for CD44 signaling in breast cancer.

Apart from specific genes and signaling pathways that are dysregulated in cancer metastasis, non-coding RNAs, and microRNAs (miRs) in particular, play a pivotal role since they can be exploited therapeutically as they can be chemically synthesized and prepared as lentiviral particles or loaded into liposomes for anti-cancer therapeutic interventions (1). Original research by Ran et al. investigated the involvement of miR-23 in multiple myeloma, as it was predicted by the online tool miRDB as a candidate miR targeting urokinase plasminogen activator (uPA), a fundamental protease in cancer cell invasion. UPA expression was found to be significantly upregulated in multiple myeloma with increasing disease severity. Also, overexpression of miR-23 in cell lines and patient-derived cells inhibits uPA expression and cancer cell invasion both *in vitro* and *in vivo* in a nude mouse model. Notably, Luo et al. reveal an interesting role for another miR in regulating cancer cell metastasis. Specifically, they assessed miRNA-145-5p expression in bone metastatic prostate cancer cells, non-metastatic cells, as well as patient tissue samples and found miRNA-145-5p levels were downregulated in prostate cancer bone metastasis. MiRNA-145-5p was also shown to inhibit cell proliferation, and epithelial to mesenchymal transition (EMT) as well as the expression of basic growth factors in bone metastatic prostate cells while Transforming Growth Factor-β2 was predicted to be its target gene. Along the same line, Alves and Geraldo identified MiR-495-3p from an *in silico* target prediction and gene enrichment analysis as one of the miRs belonging to the DLK1-DIO3 region, found on the long arm of chromosome 14 and known to host the largest miR cluster in the human genome, which was previously shown to be downregulated in papillary thyroid carcinoma. In this study, miR-495-3p expression was assessed in a cell-line panel showing reduced expression in correlation to the degree of differentiation while its loss during papillary thyroid carcinoma development plays an important role in its progression.

Another approach was employed by Li et al. that uses single-cell RNA sequencing for transcriptome profiling of individual cancer cells addressing heterogeneity at the single-cell level. They found a cluster of malignant epithelial cells with EMT and identified a hyperproliferative gene signature, which includes a family with sequence similarity 83 member D (FAM83D) malignant epithelial cells that were strongly associated with cancer-related pathways. FAM83D was shown to promote ovarian cancer progression as its downregulation in ovarian cancer cells reduced proliferation, migration, and invasion and increased cisplatin sensitivity. Moreover, binding analysis experiments revealed that FAM83D can be targeted by miR-138-5p, which can significantly reverse ovarian cancer cell migration, invasion, and EMT. Bao et al. aimed to develop a mutation-based gene-signature-established on the clinical score system to improve clinical prognosis. To that end, tissues from 144 patients with colorectal liver metastases were analyzed with next-generation sequencing and genomic alterations identified. They also tested the predicting efficiency and found it better than other scoring systems in identifying high-risk colorectal liver metastases patients who may benefit from personalized treatment.

Emerging therapies against metastasis is the focus of the review by Du et al. These authors discuss cancer progression and metastasis in osteosarcoma and review available treatments and clinical trials. They outline cell wide changes that contribute to tumor progression and metastasis describing the complex molecular and genetic alterations driving osteosarcoma metastasis.

In summary, we provide reviews and original research to describe key components involved in driving cancer metastasis that we anticipate will contribute to the overall understanding of cancer progression with the goal to better understand and target the metastatic phenotype.

## Author contributions

MM: Conceptualization, Writing – original draft, Writing – review & editing. VG: Conceptualization, Writing – original draft, Writing – review & editing.
